# Functional analysis of the rodent CK1^tau^ mutation in the circadian clock of a marine unicellular alga

**DOI:** 10.1186/1471-2121-14-46

**Published:** 2013-10-15

**Authors:** Gerben van Ooijen, Sarah F Martin, Martin E Barrios-Llerena, Matthew Hindle, Thierry Le Bihan, John S O'Neill, Andrew J Millar

**Affiliations:** 1 SynthSys, University of Edinburgh, Waddington Building, The King's Buildings, Mayfield Road, Edinburgh EH9 3JD, UK; 2 Institute for Molecular Plant Sciences, University of Edinburgh, Rutherford building 1.02A, The King's Buildings, Mayfield Road, Edinburgh EH9 3JR, UK; 3 Institute of Structural and Molecular Biology, University of Edinburgh, Edinburgh, UK; 4 MRC Laboratory for Molecular Biology, Cambridge, UK

**Keywords:** Casein Kinase 1, Circadian clock, Minimal model, *Ostreococcus tauri*, Quantitative mass spectrometry, Phospho-proteomics, Bioinformatics

## Abstract

**Background:**

Casein Kinase 1 (CK1) is one of few proteins known to affect cellular timekeeping across metazoans, and the naturally occurring CK1^tau^ mutation shortens circadian period in mammals. Functional conservation of a timekeeping function for CK1 in the green lineage was recently identified in the green marine unicell *Ostreococcus tauri*, in spite of the absence of CK1's transcriptional targets known from other species. The short-period phenotype of CK1^tau^ mutant in mammals depends specifically on increased CK1 activity against PERIOD proteins. To understand how CK1 acts differently upon the algal clock, we analysed the cellular and proteomic effects of CK1^tau^ overexpression in *O. tauri*.

**Results:**

Overexpression of the CK1^tau^ in *O. tauri* induces period lengthening identical to overexpression of wild-type CK1, in addition to resistance to CK1 inhibitor IC261. Label-free quantitative mass spectrometry of CK1^tau^ overexpressing algae revealed a total of 58 unique phospho-sites that are differentially responsive to CK1^tau^. Combined with CK1 phosphorylation site prediction tools and previously published wild-type CK1-responsive peptides, this study results in a highly stringent list of upregulated phospho-sites, derived from proteins containing ankyrin repeats, kinase proteins, and phosphoinositide-binding proteins.

**Conclusions:**

The identical phenotype for overexpression of wild-type CK1 and CK1^tau^ is in line with the absence of critical targets for rodent CK1^tau^ in *O. tauri*. Proteomic analyses reveal that two thirds of previously reported CK1 overexpression-responsive phospho-sites are shared with CK1^tau^. These results indicate that the two alleles are functionally indiscriminate in *O. tauri*, and verify the identified cellular CK1 target proteins in a minimal circadian model organism.

## Background

Cellular life on Earth has evolved circadian timekeeping to enable the anticipation of predictable daily changes in exposure to light and temperature, originating from our planet's approximately 24-hour rotation around its axis. Many high-order organismal processes are rhythmic, such as sleep, wakefulness, and alertness in animals, leaf movement in plants, and reproduction in fungi and certain algae. The basis for these rhythms is a strong cellular circadian organisation that includes rhythmic transcription of such a large set of genes that no cellular process appears to be unaffected by circadian rhythmicity. At the core of this daily transcriptional reprogramming lies a set of clock genes that regulate their own expression directly or indirectly, by a complex circuitry of transcriptional feedback loops [1,2]. The organisational logic of these transcriptional/translational feedback mechanisms (TTFLs) is broadly shared among diverse species, and for many years were thought to constitute the sole basis of circadian timekeeping in eukaryotes, although the identity of oscillating genes bare no similarity across kingdoms. Therefore, it was surprising that the intricate molecular motions of cellular TTFL systems across diverse species were found to be tuned by conserved regulator proteins such as assorted chaperones and enzymes that mediate post-translational modification [3-6]. This functional conservation stretches far along the branches of the tree of life, as the same set of regulator proteins are known to affect timekeeping in humans, rodents, *Drosophila*, and *Neurospora* and more recently were shown to affect timekeeping in the unicellular marine alga *Ostreococcus tauri*[7]. These results might reflect divergent evolution of cellular timekeeping from a common basis whereby TTFL mechanisms have been recruited in an organism- and tissue-dependent manner, appropriate to cellular function [8]. This idea is supported by the identification of rhythms in the oxidation state of peroxiredoxin (PRX) proteins in *O. tauri*[7], which are conserved in mammalian cells [9], as well as in other eukaryotes and certain prokaryotes [10]. Remarkably, under certain conditions (*O. tauri* under constant darkness, isolated human erythrocytes), the redox oscillations reported by PRX oxidation are observed to persist independently of TTFL rhythmicity, but appear to be tightly coupled with them under normal conditions [10,11].

Among the broadly conserved modifier proteins with clock function across taxa is Casein Kinase 1 (CK1)[12]. Several isoforms of CK1 are known to affect clocks in animal [13-16] and fungal model species [17]. Recently, the first identification of CK1 activity being also relevant to timekeeping in the green lineage was reported [18]. Functional conservation of CK1 in the *Ostreococcus tauri* clock goes some way to demonstrate that ubiquitous post-translational modifier proteins are indeed mechanistic components of the cellular clock. The target proteins for CK1 in the TTFLs across different kingdoms [8] are not broadly conserved. The first clock-relevant CK1 target was identified in animals; the PERIOD (PER) proteins. Rhythmic phosphorylation of PER by CK1 regulates PER stability as well as nucleocytoplasmic shuttling [15,19-22]. In Neurospora, CK1 rhythmically phosphorylates FREQUENCY (FRQ), resulting in its degradation [17]. For both animal PER and fungal FRQ, their cognate TTFL activator protein is also directly CK1-responsive; the CLOCK/BMAL complex in animals [23], and the white collar complex in Neurospora [24]. A big question thus remains how proteins like CK1 affect timekeeping across species if the TTFL target proteins that were identified in either species are not conserved between them.

The naturally occurring hamster CK1ϵ mutation *tau*[25,26] exhibits a period-shortening phenotype that was recently translated to mouse [27]. This period phenotype is a consequence of a gain-of-function mutation in the active site of CK1ϵ (R178C) resulting in increased phosphorylation of PER protein [28,29], leading to its more rapid degradation. Interestingly, the increased activity of CK1^tau^ is specific for PER and not observed for other targets. For that reason we incorporated the equivalent mutation (R200C) into an *O. tauri* CK1 overexpression construct to test whether this allele would impact on circadian phenotype indiscriminately from the wild-type CK1 allele previously published [18], or whether there would be a substrate similarly recognised as PER. This phenotype includes long-period rhythmicity, reduced sensitivity to CK1 inhibitor IC261, and a trend towards increased protein phosphorylation in the broad phospho-proteome as well as in predicted CK1 target sites. Comparison between these wild-type and CK1^tau^ allele overexpression lines revealed a close correlation on all phenotypes tested. These results validate CK1 target proteins in this minimal circadian system. Many of these target proteins are broadly conserved and should offer a rich resource to inform further studies on CK1 activity in any organism with a circadian clock.

## Results and discussion

### Affected residue in *tau* mutant hamsters is invariant across species

**Figure 1:**
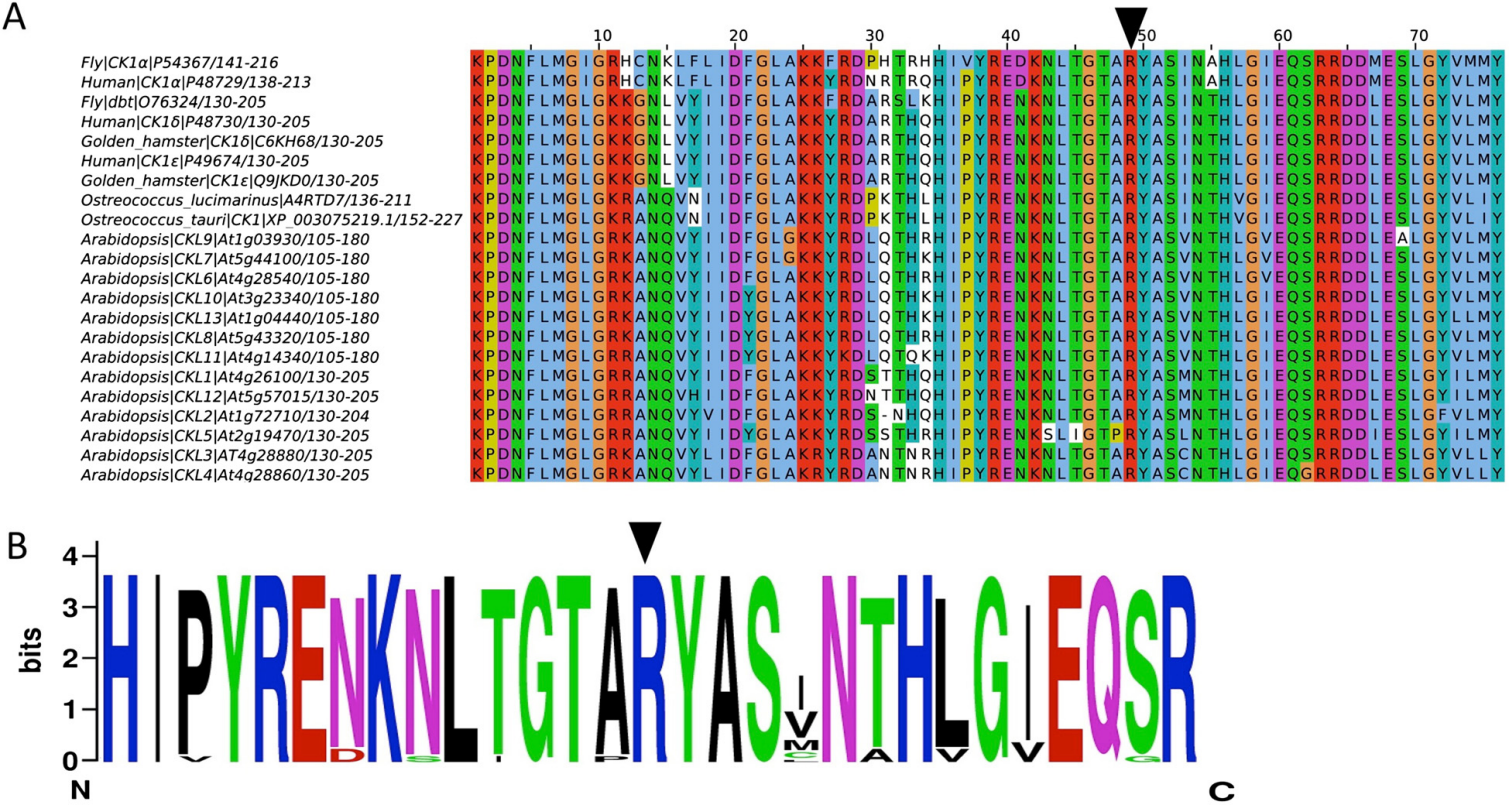
**Evident conservation of the tau mutant region across species. A)** Multiple sequence alignment of part of the kinase domain of CK1 isoforms from circadian model organisms. An arrowhead indicates location of the tau mutant from Syrian hamster. **B)** Consensus logo generated from CK1 isoforms of circadian model organisms, for the region around the tau mutation.

To identify whether the single amino acid altered in the hamster *tau* allele is present in *O. tauri* CK1, protein sequences of clock-relevant CK1 isoforms from diverse model organisms were aligned (Figure 1A). The region around the *tau* mutation [25] is highly conserved, as indicated by the consensus logo generated from the alignment (Figure 1B) implying a vital cellular function for this domain. We found that the arginine itself is invariant across all CK1 sequences tested, and in *O. tauri* corresponds to R200.

### Overexpression of CK1^tau^ induces long-period rhythms

**Figure 2:**
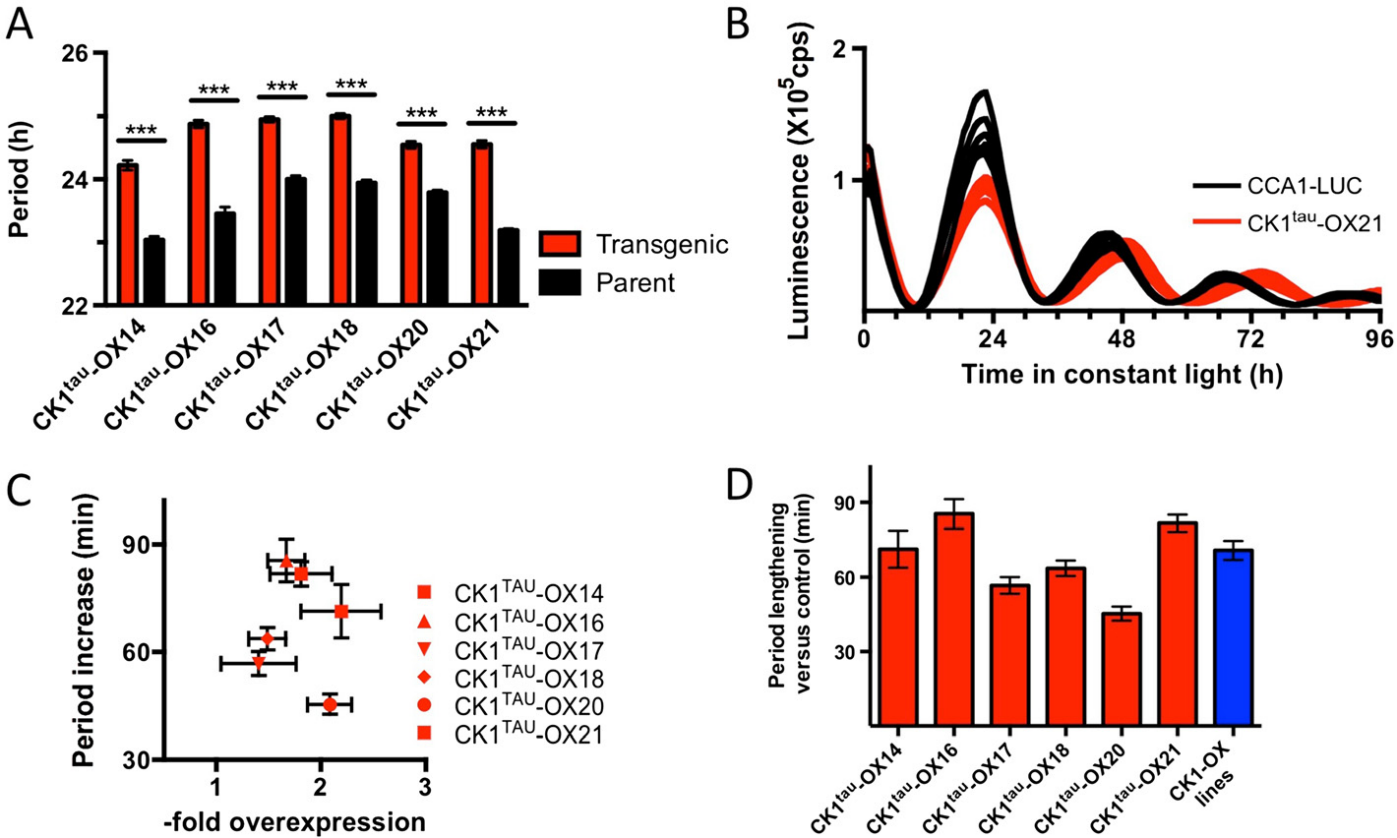
**Overexpression of CK1**^**tau **^**induces long period rhythms. A)** Free-running period was analysed in 6 independent transgenic lines overexpressing CK1^TAU^. Lines were compared against parent line CCA1-LUC in the identical plate position to the transgenic lines. In all cases, a significantly (p < 0.001) long circadian period was observed. **B)** Examples of traces of overexpression line CK1^TAU^-OX21 (red) compared to the parent line (black) in free-running conditions. **C)** Period increase (as in panel **A**) plotted against overexpression (densitometry of immunoblots, n = 3, as described in the Methods section), showing that in all transgenic lines, overexpression of the tau allele is associated with long period rhythms. **D)** Subtracted period lengthening of the six CK1^tau^ mutant lines (red bars) compared to the parent line. Combined data of six previously published [18] independent overexpression lines of the wild-type CK1 allele were plotted for comparison (in blue).

To test the effect of mutating this arginine would be, we made the analogous *tau* mutation R200C in an overexpression construct of CK1 previously used to prove conserved clock function for CK1 in *O. tauri*[18]. Similar to the previous study, transgenic lines were generated that overexpressed CK1^tau^ in cells carrying a rhythmically luminescent reporter (CCA1-LUC). After verification of transgene expression, 6 independent CK1^tau^ overexpression lines were selected and compared to the parent line. In all cases, a statistically significant long-period phenotype was observed (Figure 2A,B), associated with an approximately 2-fold overexpression level (Figure 2C). The period lengthening that was observed upon overexpression of CK1^tau^ was in the same range as that resulting from overexpression of the wild-type CK1 allele (Figure 2D) averaged for 6 independent overexpression lines previously published [18]. This result shows that the effects of both alleles on period lengthening are very similar.

### CK1^tau^ overexpression decreases the effects of CK1 inhibitor IC261

**Figure 3:**
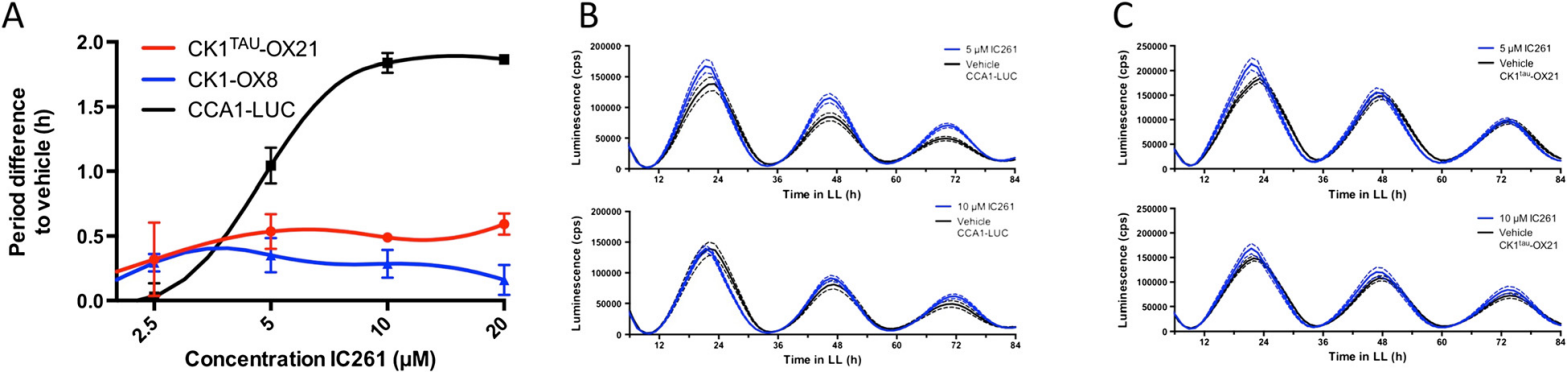
**Reduced sensitivity to CK1 inhibitor IC261 in CK1**^**tau**^**-OX21. A)** Dose–response curves of free-running period lengthening of CCA1-LUC (black line), CK1-OX8 (blue line), and CK1^tau^-OX21 (red line). CK1-OX8 results reproduced from [18] for direct comparison. **B-C)** For relevant IC261 concentrations, the averaged raw data (n = 8) is provided for CCA1-LUC parent cells **(B)** and CK1^tau^-OX21 cells **(C)**. Black lines indicate the vehicle-treated controls, and blue lines represent inhibited cells. Dashed lines indicate SD (n = 8).

Inhibition of CK1 with the specific inhibitor IC261 was previously shown to lengthen period in *O. tauri* by approximately two hours [18]. Overexpression of CK1 negated this period-lengthening effect, indicating firstly that IC261 indeed targets CK1 in *O. tauri*, and secondly that CK1 overexpression increases cellular CK1 activity. To verify that this increase in kinase activity also holds true for the CK1^tau^ overexpression lines, the effect of IC261 on the CK1^tau^-OX21 line was analysed (Figure 3). An initial phase advance is observed upon IC261 treatment, followed by a dose-dependent long period rhythm in the parent line (Figure 3A, B). The period-lengthening effect of IC261 is indeed decreased in CK1^tau^-OX21 compared to the CK1-OX line (Figure 3A, C), similarly to the result obtained with overexpression of the wild-type allele (Figure 3A and [18]).

### Phospho-proteomic analysis of CK1^tau^ overexpression line

To verify whether overexpression of the *tau* mutant indeed has an indiscriminable effect on the *O. tauri* clock from wild-type CK1 overexpression, the phospho-proteome of the parent line was compared to that of the CK1^tau^ overexpressor. Protein extracts from both were trypsinised and subjected to phospho-enrichment followed by label-free mass spectrometric quantification of each individual phospho-site detected. Out of a total of 156 detected unique phospho-sites, 43 were significantly differential (~28% of all detected sites) between the parent line and the CK1^tau^-OX21 line (Additional file 1, Figure 4); i.e. showed a fold-change of over 1.5 with p < 0.05 (n = 5). The majority of these differential phosphorylation events were upregulated (37 compared to 6 downregulated sites), verifying that CK1^tau^ represents an active kinase protein, as well as providing additional candidates for conserved CK1-mediated clock regulation.

**Figure 4:**
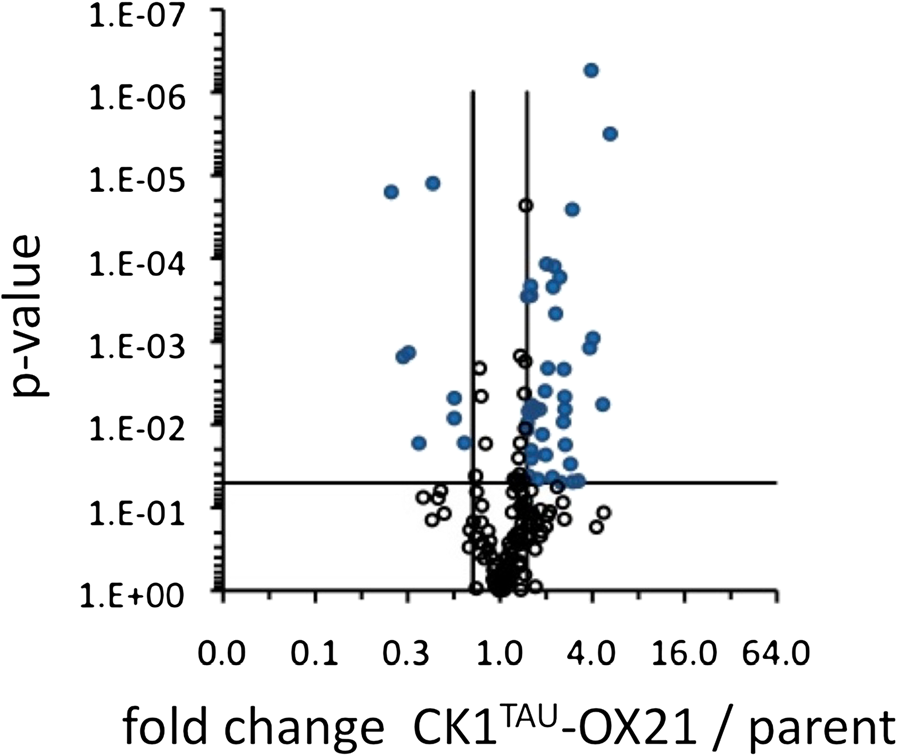
**Phospho-proteomic changes upon CK1**^**tau **^**overexpression.** Volcano plot visualising quantified phosphopeptides in the parent line and in CK1^tau^-OX21. Blue datapoints represent significantly differential phospho-sites in a pairwise comparison; black circles represent those that do not change significantly. Criteria were p < 0.05 (horizontal line) and fold changes >1.5 or <0.67 (vertical lines). The identities of sites can be found in Additional file 1.

### Strong correlation between CK1- and CK1^tau^-responsive phospho-sites

**Figure 5:**
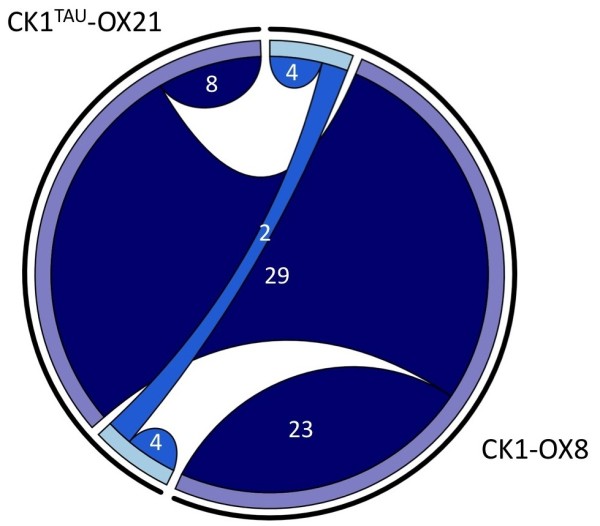
**Overlap between phospho-sites differential upon CK1**^**tau **^**and CK1 overexpression.** Chord diagram showing the numbers of identified phospho-sites whose abundance significantly differs from the parent line (>1.5- or <0.67-fold change, p < 0.05, n = 5) in phospho-proteomic analysis described here (for CK1^tau^-OX21) and in ([18], for CK1-OX8). Shared chords represent overlap between the two data sets. Upregulated peptides are represented in dark blue, downregulated peptides in lighter blue.

Combination of the results presented here with the publicly available results of label-free phospho-proteomic analysis of the wild-type CK1 allele [18] could provide evidence whether the CK1-responsive events observed are reproducible, true targets. When the significantly differential results from Additional file 1 were plotted against those published for wild-type CK1, we observed that although the total number of phospho-sites observed for CK1^tau^-OX21 was slightly lower, a majority of the upregulated sites (29 out of 37) and a third of the down-regulated sites (2 out of 6) were shared between both sets of results (Figure 5). This striking similarity between overexpression of two distinct constructs indicates that the *tau* mutation indeed is not functionally different from the wild-type allele in the *O. tauri* genetic background, and that the coverage of the phospho-proteome in this minimal circadian model organism is exceptionally high using the methods described here and previously [18]. Ultimately, the observed overlap increases confidence that the identified CK1 responsive sites are physiologically relevant protein modifications.

### Site prediction plus proteomic analyses reveal a stringent list of CK1-responsive proteins

**Figure 6:**
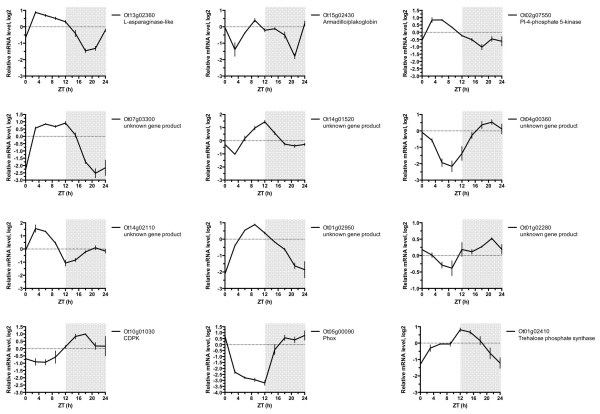
**Diurnal regulation of transcripts for parent proteins.** Micro-array data from a publically available study [30] reveals diurnal expression profiles for all the transcripts encoding parent proteins from which CK1-responsive phospho-sites were identified that were part of a predicted CK1 target site.

A second independent line of evidence supporting this interpretation comes from the site prediction results (Additional file 1). Among the phospho-sites upregulated upon CK1^tau^ overexpression, a significant overrepresentation (p = 1×10^-5^) of predicted human CK1ϵ sites was observed (13 sites on 11 proteins), further substantiating the biological activity and specificity of CK1^tau^. Strikingly, a total of 12 out of 13 predicted CK1 target sites (on 10 proteins) upregulated upon CK1^tau^ overexpression are shared with the dataset acquired with the wild-type CK1 allele (Table 1, [18]). Compared with only three predicted CK1 sites (on three proteins) that are upregulated in only one of the overexpression lines, this result indicates that this list consists of *bona fide* CK1 targets. Of the 13 genes encoding the parent proteins for the phospho-sites that were upregulated upon overexpression of both or one CK1 allele, 12 are present in publically available micro-array data from *O. tauri*[30]. Interestingly, all 12 exhibit diurnal expression profiles (Figure 6).

**Table 1:** List of predicted CK1 sites present among identified CK1-responsive phospho-sites

**Protein description**		**Site sequence**	**Gene ID**	**Closest human relative**	**E**	**mi**	**Accession**	**Alters Hs clock?**	**Rhythmic in *Mm*?**
	***Upregulated in both lines:***							**	***
1	L-asparaginase-like	S(p)LGLLPGPTK	Ot13g02360	L-asparaginase	1e-35	34%	AAH35836.1	Yes (τ, A)	Yes (l, a, ba, ag)
2	Armadillo/plakoglobin	AAS(p)AEIASDYVATPGGSR	Ot15g02430	Uridine-Cytidine Kinase 1-like	3e-20	32%	EAW75186.1	-	Yes (ba, a, d)
3	PI-4-phosphate 5-kinase	LRSS(p)VANVTAFATEEPL	Ot02g07550	PI-4-phosphate 5-kinase	2e-06	23%	AAC50916.1	Yes (τ, A)	Yes (h, k, a, n)
4	Ankyrin repeats protein	AMQRGSS(p)LLDLQSADGGTPAMSAAAHSYGDVLQYLIEK	Ot08g01070	Ankyrin repeat domain 17	2e-05	38%	AAH43394.1	Yes (τ, A)	Yes (bs, l, n, h, s)
5	unnamed protein product	ASTLNDST(p)ADDGNVVR	Ot07g03300	Ankyrin/Armadillo repeats protein	0.014	24%	EAX10893.1	-	-
6	unnamed protein product	EAFGDAS(p)DDDAFSPR	Ot14g01520	Poliovirus receptor-related 4	1.6	32%	BAG61075.1	Yes (τ, A)	Yes (n, d)
7	unnamed protein product	ET(p1)KT(p2,3)LAELMS(p3)INMGA*	Ot04g00360	RNF181	1.8	42%	CCQ43543.1	-	-
8	unnamed protein product	GGQEGS(p)PSKSLSSPK	Ot14g02110	No similarity found	N/A	N/A	N/A	N/A	N/A
9	unnamed protein product	ALEDES(p)PVAVVKEK	Ot01g02950	No similarity found	N/A	N/A	N/A	N/A	N/A
10	unnamed protein product	TKDAAEAS(p)DEDVVATR	Ot01g02280	No similarity found	N/A	N/A	N/A	N/A	N/A
	***Upregulated in *** **tau *only:***								
11	CDPK	SES(p)FAILTEAAK	Ot10g01030	Death-associated protein kinase 3	2e-37	31%	NP_001339.1	Yes (τ, A)	Yes (d, lu, hy)
	***Upregulated in CK1-OX8 only:***								
12	Phox	AIS(p)PAPEER	Ot05g00090	Sorting nexin 1, phox domain	4e-13	22%	NP_003090.2	Yes (τ, A)	Yes (d, l)
13	Trehalose-phosphate synthase	PADGST(p)PESPPRR	Ot01g02410	H+ transporting ATPase	5.8	35%	EAW99791.1	Yes (τ)	Yes (lu, k, h)
	***Not differentially regulated in either overexpression line:***								
14	putative TAF6 RNA polymerase	GT(p)TPDDDIGDAAAAHAPNVAVAETHV	Ot13g00540	TAF6 RNA polymerase II	4e-65	34%	EAW76588.1	Yes (τ, A)	Yes (l, s, p, ag)
15	unnamed protein product	APAGAKPGITLPSNPFAAKPAT(p)KATPAAK	Ot14g01370	Unknown protein	2e-38	40%	AAH53854.1	-	-
16	unnamed protein product	FGIVDGS(p)ASTETPETFVK	Ot08g03890	Spondin 1	0.36	25%	AAH19825.1	Yes (τ)	Yes (lu, n, d)
17	unnamed protein product	ALASDS(p)EDDERPR	Ot04g04920	Importin beta	1.1	38%	NP001263382.1	-	-
18	unnamed protein product	TAS(p)PMTSPMASPSPAD	Ot17g01690	2,3-bisphosphoglycerate mutase	9.2	33%	BAD92281.1	Yes (τ, A)	Yes (n, sm, p, d)
19	unnamed protein product	SAS(p)YDSLLGAVPASTFPRPIPLAEMVR	Ot15g00280	No similarity found	N/A	N/A	N/A	N/A	N/A

List of CK1 sites based on site prediction that are present in the phospho-preoteome observed in this study and [18]. Parent proteins are listed for each peptide, along with their closest homolog in the human genome. Results are listed based on 1) their presence in significantly differentially regulated sites, and 2) Goodness of homology to closest human ortholog identified by BLAST searches. E values (E) and Maximum Identity (MI) are based on NCBI Blast. *This peptide is identified in three different phosphorylation states (p1, p2, p3), and all three are upregulated upon overexpression of either allele. ** [34], effects on period (t) and/or amplitude (A) upon knock-down in human cells. *** [36-41], rhythmic expression in following mouse cell types: liver (l), aorta (a), brown adipose (ba), adrenal gland (ag), distal colon (d), heart (h), kidney (k), NIH3T3 (n), brain stem (bs), SCN (s), lung (lu), hypothalamus (hy), pituitary (p), skeletal muscle (sm).

The most stringent set of candidates, upregulated in both CK1 overexpression lines, and additionally part of a predicted CK1 target sequence, consist of 12 *O. tauri* peptides from 10 proteins. Unfortunately, 6 peptides stem from unknown and unannotated proteins meaning that either no significant homology is found with any other known protein, or that the gene models for these proteins are incorrect. To test whether these 10 stringently verified CK1-responsive target sites might relate to evolutionarily conserved clock-relevant target proteins, all *O. tauri* parent protein sequences were compared to the human proteome. Half of these returned significant (E value <0.02) homology (Table 1) and of these 5 human proteins, two are kinases (Uridine-Cytidine Kinase 1-like and PI4P5-kinase) and three are proteins containing ankyrin repeats (L-asparaginase and Ankyrin repeat proteins).

Such kinases are potentially involved in mediating functional cellular circadian outputs and thus might be expected to be regulated in turn by a hub kinase like CK1 [12]. This observation also agrees with the prevailing view of richly interconnected kinase networks, rather than the linear signal transduction pathways in the older literature, and could go some way to explaining how CK1 inhibition as well as overexpression induces a long-period phenotype, as both treatments may be expected to have pleiotropic downstream effects on multiple pathways, thereby compromising cellular timekeeping function.

Ankyrin repeats are solenoid protein domains involved in protein-protein interactions [31,32]. Although more common in eukaryotes, these do exist across all domains of life [33]. Ankyrin repeats are identified in proteins involved with transcriptional regulation, signal transduction, cell cycle regulation, and ion transport [32]. As 3 out of the 5 most stringent hits resulting from this and a previous study contain ankyrin repeats, it is likely that CK1 regulates these protein interactions and their signalling activity. The broad conservation of ankyrin repeats and kinase proteins implies that the evolutionary basis of conserved CK1-mediated timekeeping mechanisms could rely on these classes of proteins.

To further substantiate that the protein in listed in Table 1 contain clock-relevant CK1 targets, we looked for the closest human homologs in a genome-wide microRNA screen [34] for clock-relevant genes in human cells, using BioGPS [35]. Ten out of fifteen of the proteins listed in Table 1 have an effect on clock amplitude (A) and/or period (t) when knocked down in human cells (Table 1), compared to 20 to 25% hit rate in the original paper. In addition, transcription of most of the closest mouse homologs was found to be clock-regulated in a wide range of tissues (Table 1, [36-41]). Combined, these results indicate a role in sustaining wild-type circadian rhythms across taxa for the CK1 targets identified here in *O. tauri*.

## Conclusions

The results presented here and in previous work provide solid proof for the notion that CK1 targets can be conserved across domains of life, and include important signalling proteins. Mammalian homologs of the targets identified here are involved in cellular timekeeping, and future work should investigate the functional cellular and chronobiological consequences CK1 activity on these conserved target proteins.

## Methods

Unless otherwise stated, chemicals were obtained from Sigma-Aldrich.

### Culturing and imaging

*Ostreococcus tauri* culturing and imaging was performed as described previously [7,42]. The parent *Ostreococcus* line used to generate the CK1^tau^ overexpression lines is described in [43]. Circadian period analyses were calculated using the mfourfit algorithm on BRASS3 [44]. Pharmacological experiments were performed as described in [18] with a replicate number of 8. Comparable vehicle treatments were subjected to identical treatments and grown and imaged in the identical well of a dummy plate.

### Construction of transgenic materials

CK1 was amplified from genomic DNA and cloned as described in [18]. The tau mutation R200C was incorporated using circular mutagenesis using the following oligonucleotides: gacgggaacggcgTGTtacgcgagtatcaacacg, cgtgttgatactcgcgtaACAcgccgttcccgtc. Vectors were verified using standard sequencing. Transgenic lines were generated as described previously [42,43], and presence of the tau mutation in a genomic DNA extraction was confirmed using control oligonecleotides gcgtgttgatactcgcgtaACA and gcgtgttgatactcgcgtaTCT. For immuno-blotting of CK1^tau^, cell extracts were prepared, separated, and blotted as described in [11], and blocked with 0.25% BSA / 0.25% skimmed milk powder in TBST buffer. Primary antibody against human CK1δ (Sigma, SAB2104925) was used at a dilution of 1:10.000. The epitope region of 50 amino acids contains only 5 mismatches in *O. tauri* and recognises a single band at the expected size that is more abundant in the overexpression lines as indicated in Figure 2C. Densitometry of immuno-blots was described in [11].

### Phospho-proteomics

Protein extraction and phospho-proteomic analyses were performed identically to what is described in [18]. Acetonitrile and water for sample preparation were HPLC quality (Fisher, UK). Formic acid was Suprapure 98-100% (Merck, Darmstadt, Germany) and trifluoroacetic acid was 99% purity sequencing grade. LC-MS label-free quantification was performed using Progenesis 4.0 (Nonlinear Dynamics, UK) as described in [45] and [18]. Ion extraction, MSMS data searching, and Mascot searching parameters were identical to [18]. The mass spectrometry proteomics data were validated using the PRIDE converter 2 [46] and have been deposited to the ProteomeXchange Consortium  (http://proteomecentral.proteomexchange.org) via the PRIDE partner repository [47] with the dataset identifier PXD000378 and DOI 10.6019/PXD000378*.* Neutral losses of phosphoric acid typical of serine and threonine phosphorylated were validated manually in all significantly differential phospho-peptides. Where multiple occurrences of residue phosphorylation events were quantified, abundances were summed, collating all charge states, missed cuts and further modifications.

### Statistical analyses

Graphs and statistical analyses were prepared using GraphPad Prism unless otherwise stated. For Figure 1, CK1 sequences were aligned using Mafft 6 [48]. For statistical analyses on the phospho-preoteomics results, data were tested at the phosphorylation site level identically to how it is described in [18]: Abundances were arcsinh transformed to generate normal distributions. Phospho-sites with a significantly differential mean-abundance, compared to the control, were identified with a two-tailed t-test for independent samples. Within group means on raw values were calculated to determine the fold changes. Peptides with p < 0.05 and fold change ratio >1.5 between groups were defined as significantly differential.

To predict CK1 target sites, GPS 2.1 [49] was used on the v2.0 assembly of the *Ostreococcus tauri* genome [50,51]. Peptides were aligned to the reference assembly and CK1 targets were transferred. Permutation-tests were used to test if the mean fold-change of CK1ϵ targets was significantly different to the remaining peptides in the dataset. The significance of over- or under-representation of CK1 motifs was estimated using a Monte Carlo permutation test. The co-occurrence of unique residue phosphorylation events was enumerated across all peptides between all statistically significant groups. The resulting co-occurrence of phosphorylation events was visualised using the chord visualization [52] from the D3 library (http://d3js.org).

### Database searches

For Table 1, closest human homologs were identified by protein blast at NCBI (blast.ncbi.nlm.nih.gov). Effects on human circadian rhythms was analysed with BioGPS ([35], biogps.org). Mouse transcriptional data was analysed using the Circadian BioGPS plugin to the circadian expression profiles database (CircaDB, bioinf.itmat.upenn.edu/circa) of the Hogenesch lab. Circadian expression profiles in *Ostreococcus tauri* were analysed by microarray experiments described in [30].

## Abbreviations

BMAL: Brain and muscle arnt1-like; CK1: Casein Kinase 1; FRQ: Frequency; MS: Mass spectrometry; NTO: Non-transcriptional oscillator; PER: Period; TTFL: Transcriptional/ translational feedback loop; WCC: White collar complex.

## Competing interests

The authors declare that they have no competing interests.

## Authors’ contributions

GvO generated materials, performed experiments, and analysed data. SFM, MBL and TLB performed phospho-proteomics and analysed data. MH performed the phylogeny and CK1 site predictions. JSO'N and AJM provided intellectual framework. GvO and AJM directed the work and designed strategy. GvO wrote the manuscript. All authors read and approved the final manuscript.

## Supplementary Material

Additional file 1 **Identified phospho-sites in the overexpression line CK1**^**tau**^**-OX21 versus parent line CCA1-LUC, with identification and quantification details plus CK1 site prediction results.** Click here for file
